# Supplementary material for: Design and Immune Profile of Multi-Epitope Synthetic Antigen Vaccine Against SARS-CoV-2: An In Silico and In Vivo Approach
Source: Vaccines (Basel). 2025 Jan 31;13(2):149. doi: 10.3390/vaccines13020149 (PMC11861798; doi:10.3390/vaccines13020149)
Supplement: Supplementary file 1 [file vaccines-13-00149-s001.zip › Suplementary Material.pdf]

Supplementary Materials

Tables attached in the Excel file

Table S1. Population Coverage.

Table S2. Cross reactivity analysis by BLASTp

Table S3. Molecular Docking Scovsint1 - TLRs

A) HLA-B*53:01 / HLA-B*51:01 / HLA-A*02:01 / HLA-A*29:02 / HLA-DRB1*13:02 / HLA-DRB1*11:01

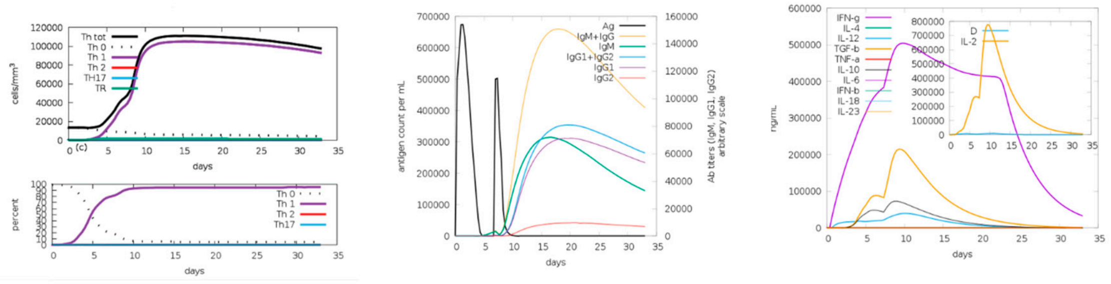

B) HLA-B*53:01 / HLA-B*51:01 / HLA-A*02:01 / HLA-A*29:02 / HLA-DRB1*11:02 / HLA-DRB1*11:21

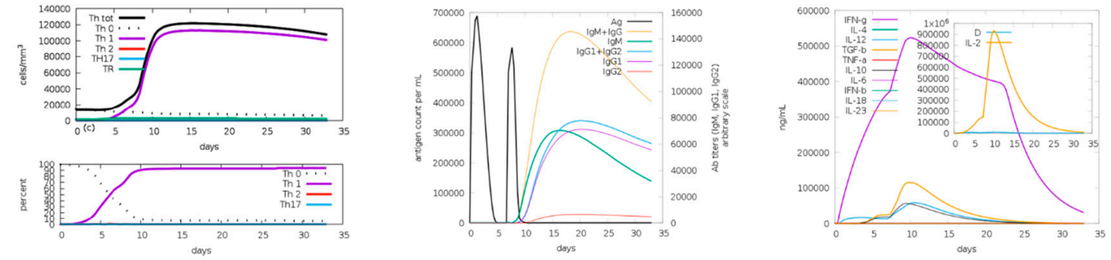

C) HLA-B*53:01 / HLA-B*51:01 / HLA-A*02:01 / HLA-A*29:02 / HLA-DRB1*13:22 / HLA-DRB1*13:04

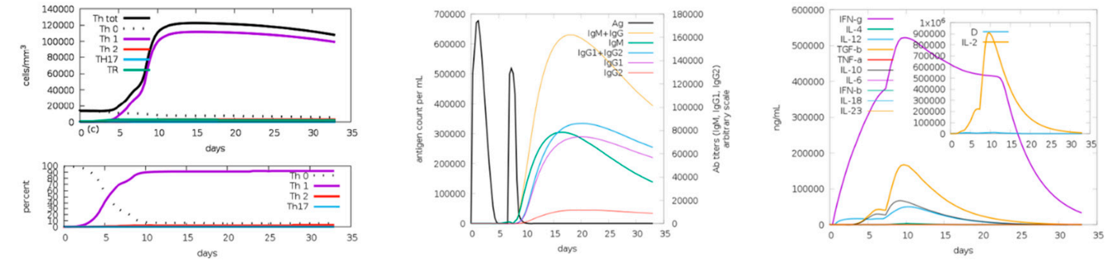

D) HLA-B*53:01 / HLA-B*51:01 / HLA-A*02:01 / HLA-A*29:02 / HLA-DRB1*08:06 / HLA-DRB1*11:28

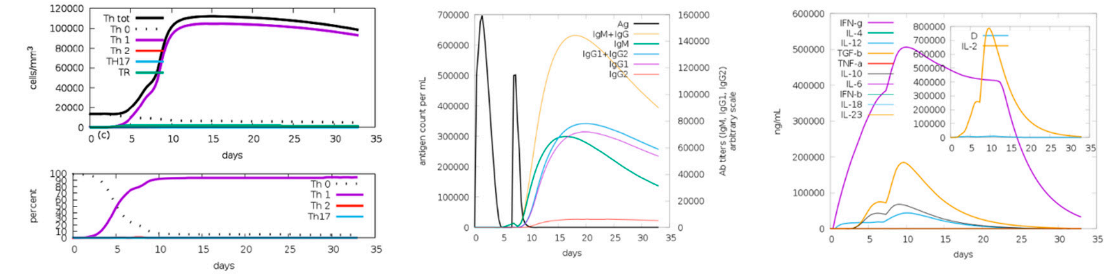

E) HLA-B*35:01 / HLA-B*15:02 / HLA-A*29:02 / HLA-A*03:01 / HLA-DRB1*01:01 / HLA-DRB1*11:04

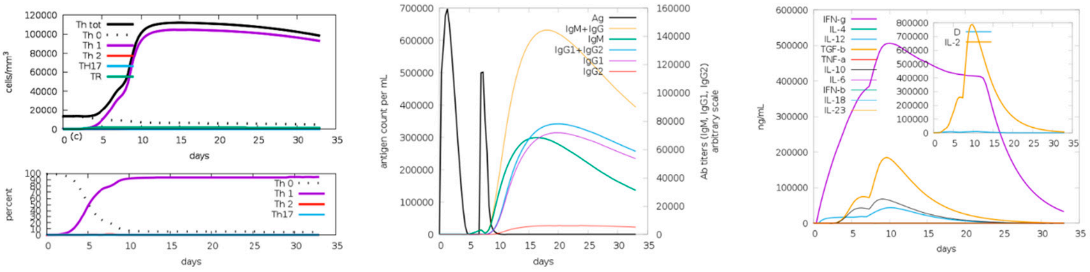

F) HLA-B*35:01 / HLA-B*15:02 / HLA-A*29:02 / HLA-A*03:01 / HLA-DRB1*13:05 / HLA-DRB1*08:04

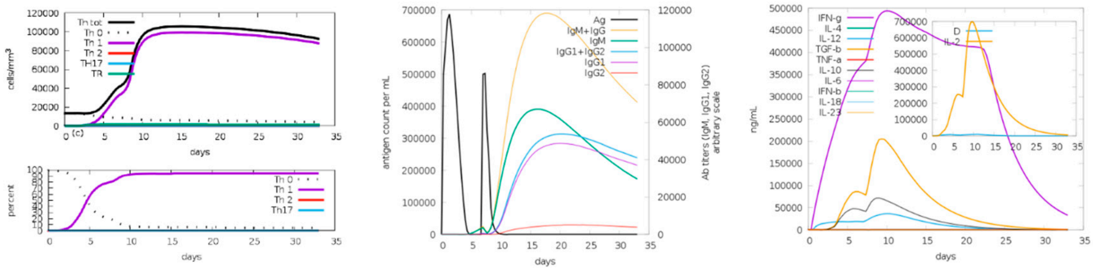

G) HLA-B*35:01 / HLA-B*15:02 / HLA-A*29:02 / HLA-A*03:01 / HLA-DRB1*11:14 / HLA-DRB1*13:23

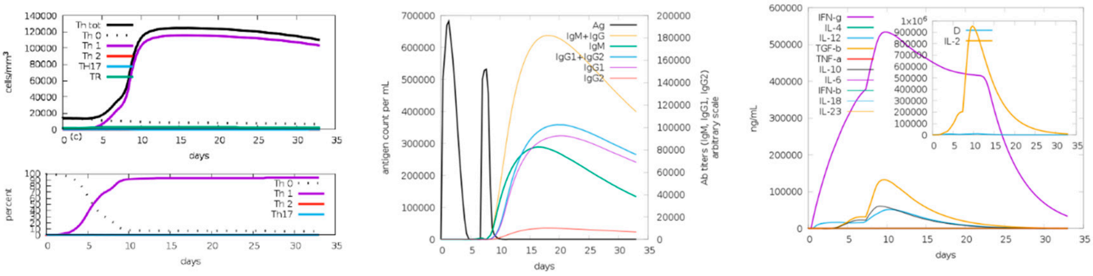

H) HLA-B*35:01 / HLA-B*15:02 / HLA-A*29:02 / HLA-A*03:01 / HLA-DRB5*01:05 / HLA-DRB5*01:01

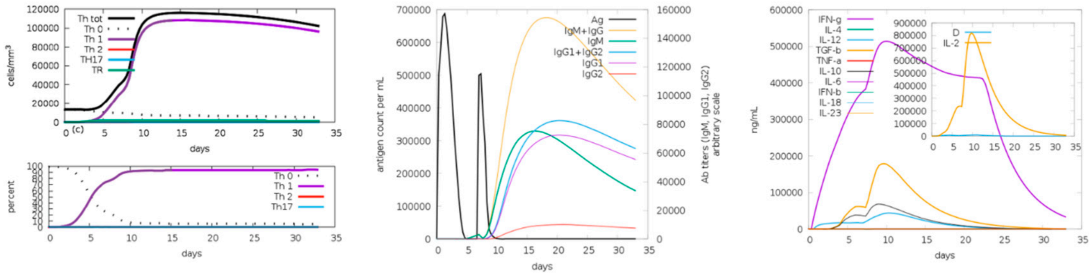

I) HLA-B*15:01 / HLA-B*35:03 / HLA-A*01:01 / HLA-A*03:01 / HLA-DRB1*11:06 / HLA-DRB1*13:11

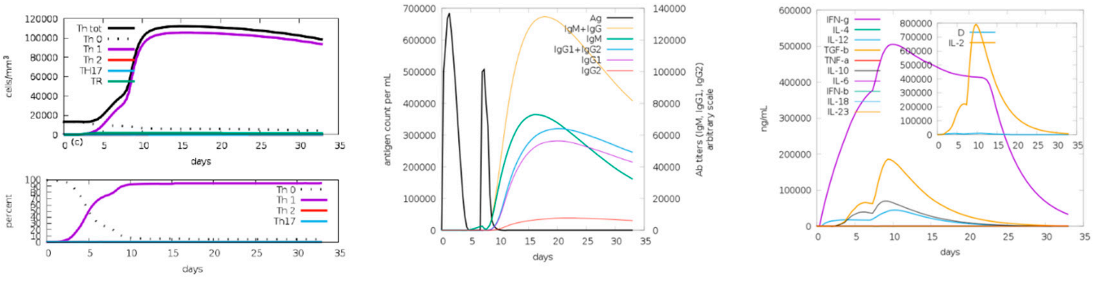

J) HLA-B*15:01 / HLA-B*35:03 / HLA-A*01:01 / HLA-A*03:01 / HLA-DRB1*11:06 / HLA-DRB1*08:04

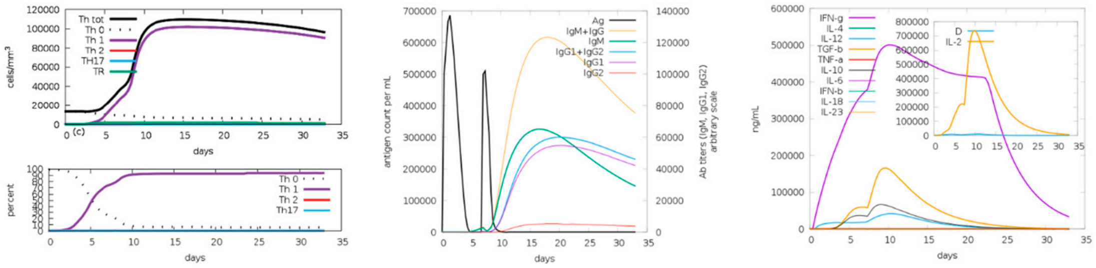

K) HLA-B*15:01 / HLA-B*35:03 / HLA-A*01:01 / HLA-A*03:01 / HLA-DRB1*13:21 / HLA-DRB1*13:07

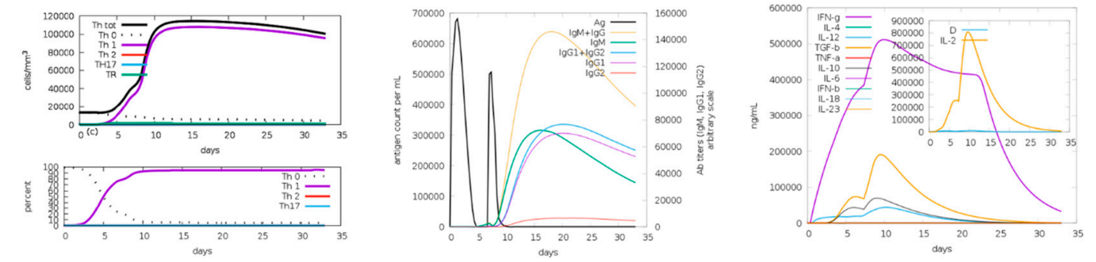

L) HLA-B*15:01 / HLA-B*35:03 / HLA-A*01:01 / HLA-A*03:01 / HLA-DRB1*11:28 / HLA-DRB1*13:05

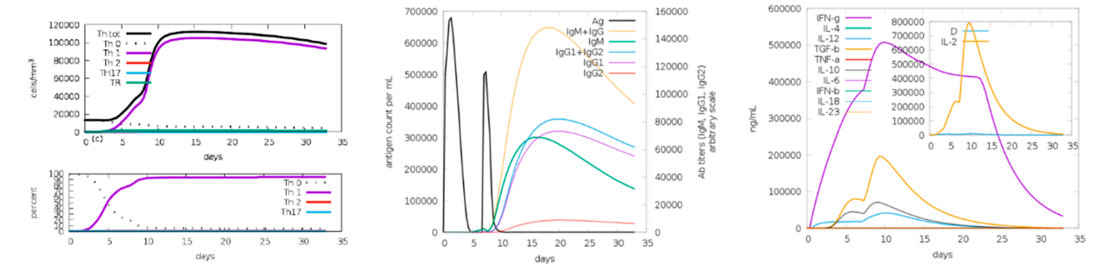

Figure S1. Immune simulation by C-IMMSIM.

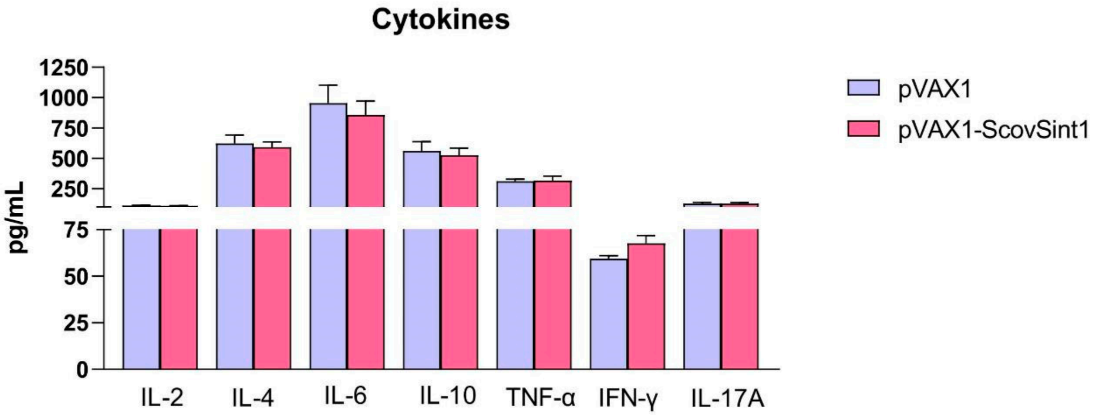

Figure S2. Cytokine dosage from blood samples 14 days after the second dose.

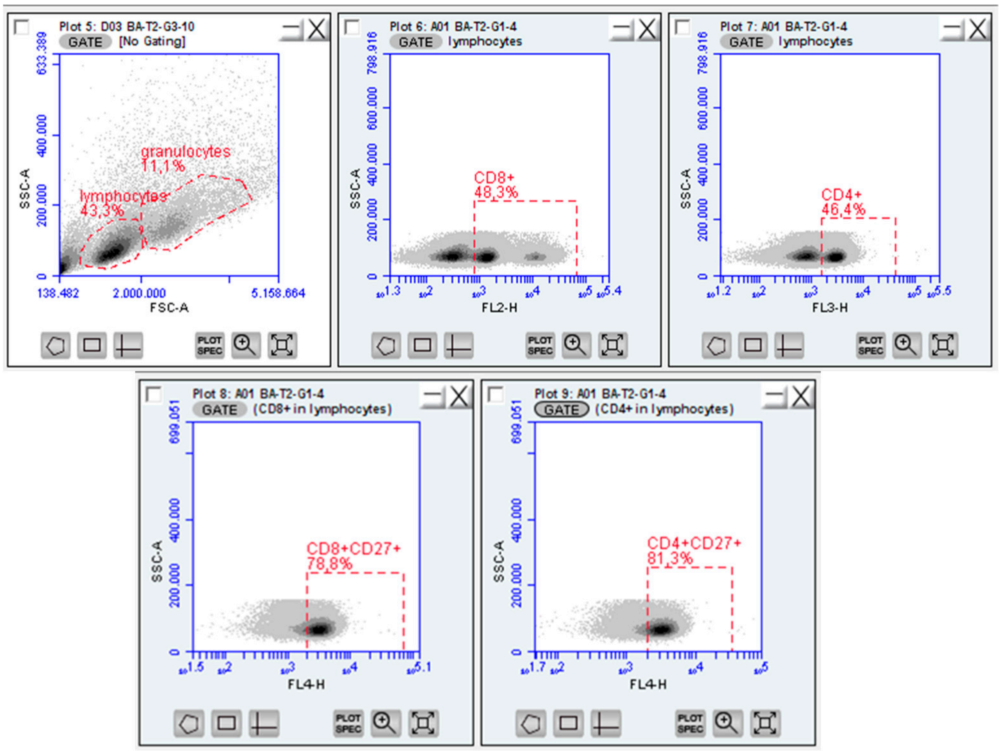

Figure S3. Gating strategy used to identify different lymphocyte subpopulations. Within the lymphocyte gate, CD8+ T cells were labeled with AntiCD8-PE and detected using the FL2-H channel. CD4+ T cells (labeled with AntiCD4-FITC). CD8+CD27+ T cells were isolated in the CD8+ gate (labeled with AntiCD27-APC) and detected by FL4-H. CD4+CD27+ T cells were isolated in the CD4+ gate (labeled with AntiCD27-APC) and detected by FL4-H.

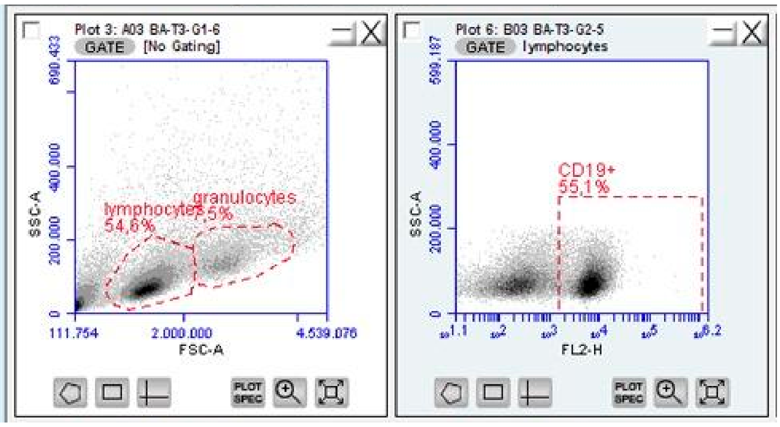

Figure S4. Gating strategy used to identify different lymphocyte subpopulations. Within the lymphocyte gate, CD19+ cells (labeled with AntiCD19-PE) were isolated and detected by the FL2-H channel.

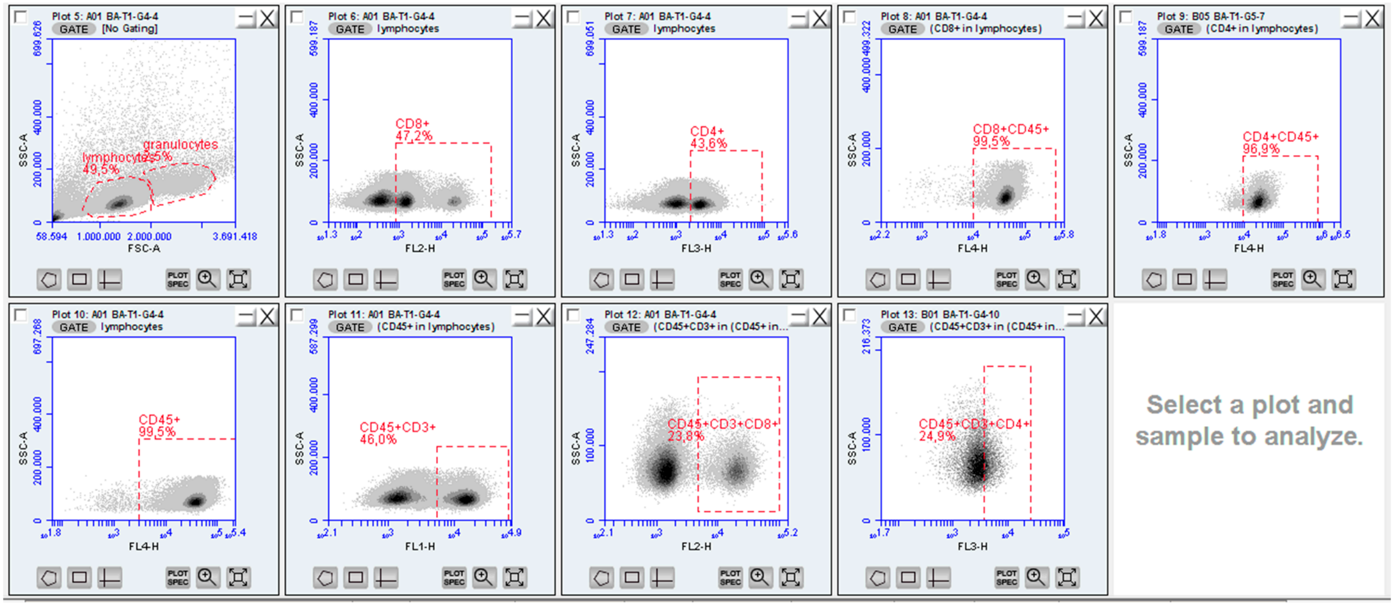

Figure S5. Gating strategy used to identify different lymphocyte subpopulations. In the lymphocyte gate, CD8+ T cells were labeled with AntiCD8-PE and detected using the FL2-H channel. CD4+ T cells (labeled with AntiCD4-FITC, detected in FL3-H). CD8+CD45+ T cells were isolated in the CD8+ gate (labeled with AntiCD45-APC) and detected by FL4-H. CD4+CD45+ T cells were isolated in the CD4+ gate (labeled with AntiCD45-APC) and detected by FL4-H. CD45+CD8+ and CD45+CD4+ were isolated from the CD45+ gate.

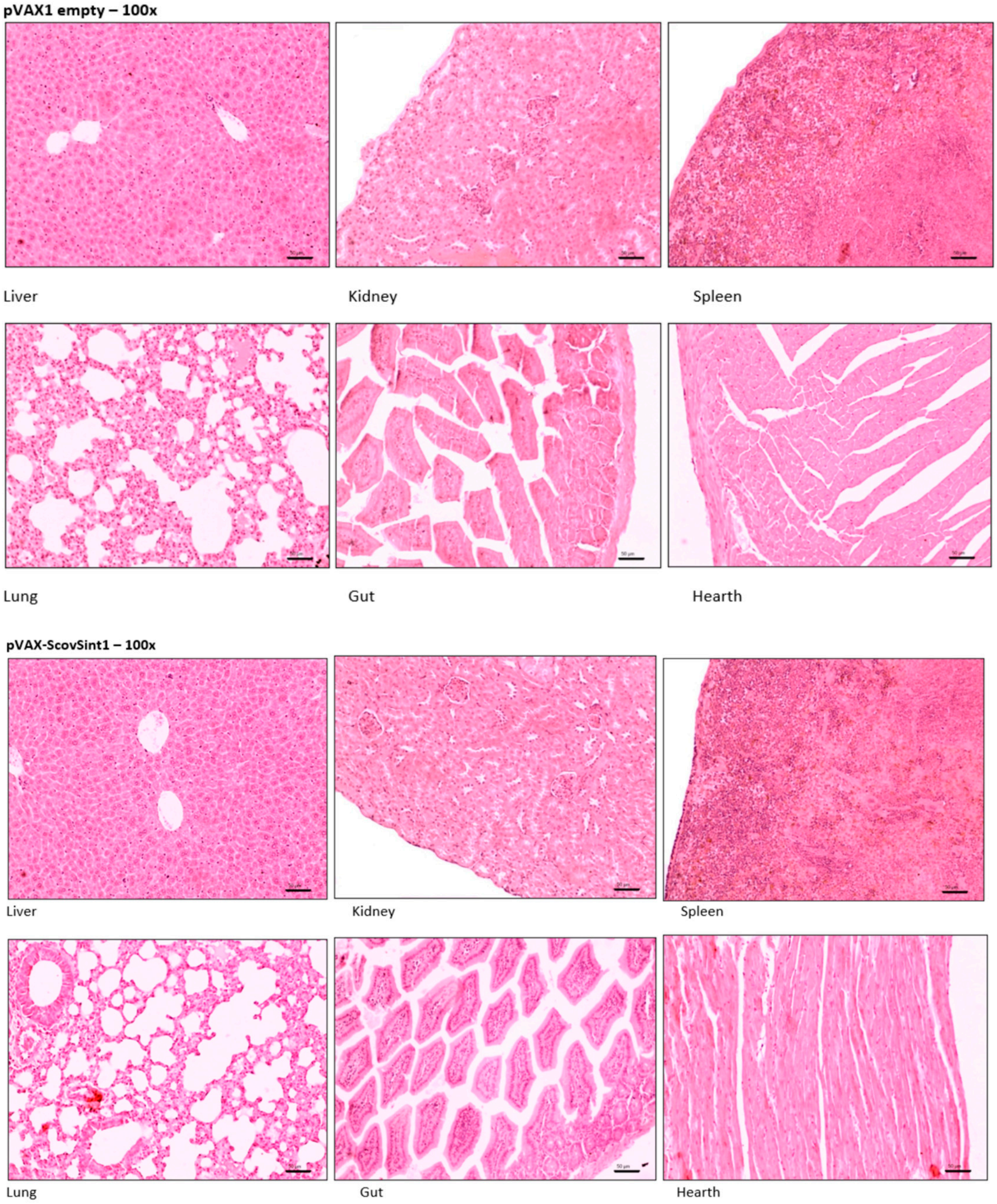

Figure S6. Representation of the histopathological samples with pVAX1 empty and pVAX-ScovSint1 in different organs. Liver: Tissue structure in pattern of normality (without reactive changes or degeneration). Kidney: Structures, including glomerular compartment, tubular, vascular and interstitial preserved. Absent changes in the Bowman space and infiltrates. Spleen: Structure preserved, without histomorphology changes, including capsule, white pulp (evident lymphoid follicles) and red pulp preserved. Absent of congestive process and reactivity. Typical macrophages. Lung: Terminal bronchioles and alveoli with characteristics of the typical breath tissue. Gut: Gut without histopathological changes in mucous, submucosal and adventitia layers. Hearth: Preserved cardiac musculature, with fibers typically distributed and without histopathological changes.
